# Biogeographic Pattern and Network of Rhizosphere Fungal and Bacterial Communities in *Panicum miliaceum* Fields: Roles of Abundant and Rare Taxa

**DOI:** 10.3390/microorganisms11010134

**Published:** 2023-01-04

**Authors:** Lixin Tian, Yuchuan Zhang, Liyuan Zhang, Lei Zhang, Xiaoli Gao, Baili Feng

**Affiliations:** 1 State Key Laboratory of Crop Stress Biology in Arid Areas, College of Agronomy, Ministry of Agriculture, Northwest A&F University, Xianyang 712100, China; 2 Chifeng Academy of Agricultural and Animal Husbandry Sciences, Chifeng 024031, China; 3 Crop Research Institute, Gansu Academy of Agricultural Sciences, Lanzhou 730070, China

**Keywords:** poso millet, rhizosphere microbes, rare and abundant taxa, co-occurrence patterns, assembly processes

## Abstract

Unraveling how microbial interactions and assembly process regulate the rhizosphere abundant and rare taxa is crucial for determining how species diversity affects rhizosphere microbiological functions. We assessed the rare and abundant taxa of rhizosphere fungal and bacterial communities in proso millet agroecosystems to explore their biogeographic patterns and co-occurrence patterns based on a regional scale. The taxonomic composition was significantly distinct between the fungal and bacterial abundant and rare taxa. Additionally, the rare taxa of bacteria and fungi exhibited higher diversity and stronger phylogenetic clustering than those of the abundant ones. The phylogenetic turnover rate of abundant taxa of bacteria was smaller than that of rare ones, whereas that of fungi had the opposite trend. Environmental variables, particularly mean annual temperature (MAT) and soil pH, were the crucial factors of community structure in the rare and abundant taxa. Furthermore, a deterministic process was relatively more important in governing the assembly of abundant and rare taxa. Our network analysis suggested that rare taxa of fungi and bacteria were located at the core of maintaining ecosystem functions. Interestingly, MAT and pH were also the important drivers controlling the main modules of abundant and rare taxa. Altogether, these observations revealed that rare and abundant taxa of fungal and bacterial communities showed obvious differences in biogeographic distribution, which were based on the dynamic interactions between assembly processes and co-occurrence networks.

## 1. Introduction

Farmland is a typical human-managed terrestrial ecosystem that contributes significantly to food security [1]. In this ecosystem, soil microorganisms are a critical link between the belowground and aboveground ecological components, showing a diverse array of taxa and diversity [2,3,4]. Clarifying the assembly processes, microbial diversity, taxonomic interactions, and distribution patterns are essential for uncovering the biogeochemistry and stability of the rhizosphere ecosystem in proso millet fields.

It is well known that soil microorganisms often include a few widely distributed abundant taxa and numerous restricted distributions of rare taxa [5]; the former taxa are considered to be essential for dissolved organic matter flux and carbon cycling [6], whereas the latter taxa, which is known as the “rare taxa”, contains a reservoir of essential functional diversity that may regulate a wide variety of ecosystem functions, substantial metabolically active lineages, and nutrient cycling [7,8,9]. Distinguishing between rare and abundant taxa from the perspective of biogeography and community assembly can help us know microbial-driven ecosystem processes and functions and is the central task of microecology [1,10]. The structures of rare and abundant taxa are influenced by distinct governing variables or ecological processes. For example, rare taxa of bacteria are more sensitive to environmental conditions, whereas abundant taxa are mainly dependent on spatial variables in marine and soil ecosystems [11,12]. Meanwhile, considering the stability of agro-ecosystem, the impacts of distance and environmental variables on rare and abundant taxa in soil might be distinct from those in aquatic ecosystems. To date, the biodiversity and assembly process of rare and abundant taxa and the main forces governing their distribution in rhizosphere fungal and bacterial communities across agroecosystems at region scales have not been clarified.

Microbe–microbe interactions play a crucial role in governing microbial communities. Investigating their co-occurrence patterns can unravel the potential roles and complex interactions of abundant and rare taxa [13,14]. The potential meaning of nodes can be assessed by studying topological features in microbial co-occurrence networks [15]. Several literature studies have revealed the different roles of abundant and rare taxa in ecologic networks, including the revelation that the role of abundant taxa is more essential than the role of rare ones [16,17,18]. However, relevant reports about the ecological networks of rare and abundant taxa, particularly in farmland rhizosphere soil based on a regional scale, is still lacking.

Proso millet (*Panicum miliaceum* L.) is a minor grain crop, which is of great significance to the adjustment of agricultural production in northern China [19]. Regulating the interactions of microbial communities, especially in the rhizosphere, to ensure healthy crop growth is a potential way of increasing proso millet productivity. Therefore, we explored the biogeographic patterns, ecological networks, and assembly processes of rhizosphere fungal and bacterial abundant and rare taxa from twelve proso millet fields in northern China. Specifically, our purpose is to investigate (i) the biogeographic patterns of rare and abundant taxa, (ii) the relative contributions of the assembly processes to each of these taxa, and (iii) their co-occurrence networks.

## 2. Materials and Methods

### 2.1. Experimental Site and Data Collection

Proso millet cv Yumi No. 2 was planted in three 20 m^2^ plots located at twelve sites across northern China (Figure S1), and was cultivated under conventional cultivation conditions. Thirty-six samples were obtained from the twelve sites (three samples per site) at the proso millet flowering period (July–August 2020). The roots of 10–15 healthy plants were obtained in each plot employing a sterilized shovel, and shaken gently to discard bulk soil, and select the soils attached to the roots about 0.2 cm, namely rhizosphere soil, and combined them into a sample. Finely samples were sieved employing 2 mm mesh sieves. A rhizosphere subsample was placed at 4 °C to measure the soil properties, namely pH, total nitrogen (TN), organic matter (OM), available potassium (AK), available phosphorus (AP), ammonium nitrogen (NH_4_^+^-N), and nitrate nitrogen (NO_3_^−^-N) (see Supplementary Material). Another rhizosphere subsample was stored at −80 °C for DNA extraction within 24 h. Furthermore, mean annual precipitation (MAP) and mean annual temperature (MAT) were gained from the China Meteorological Data Service Center (http://data.cma.cn/, accessed on 25 November 2020.).

### 2.2. DNA Extraction and Sequence Analysis

According to the statements of [20], the DNeasy 96 PowerSoil Pro QIAcube HT kit (QIAGEN, Hilden, Germany) was used to extract total DNA from 500 mg rhizosphere soil in each sample. The total DNA was submitted to Majorbio Bio-pharm Technology Co., Ltd. (Shanghai, China) for high-throughput sequencing on the MiSeq PE300 platform (Illumina, San Diego, CA, USA). More details on the sequence analysis can be observed in the supplementary information. The fungal and bacterial sequences were aligned with the Unite database (http://unite.ut.ee/index.php, accessed on 25 November 2020 version 8.0, Urmas Kõljalg, Tartu, Estonia) [21] and the Silva database (https://www.arb-silva.de/, version 138, accessed on 25 November 2020 Frank Oliver Glöckner, Bremen, Germany) [22], respectively.

The definition of abundant and rare taxa depends on the threshold of their relative abundance, with 1% indicating abundant OTUs and 0.01% indicating rare OTUs [5,8,23]. These classifications can ignore intermediate taxa (i.e., those with the relative abundance between 0.01% and 1%) and oscillating taxa (i.e., taxa that can be both rare and abundant depending on environmental conditions). In the present study, we classified all OTUs into the following six categories according to previous study [24]: (1) always abundant taxa (AAT) with the relative abundance of ≥1% in all samples; (2) conditionally abundant taxa (CAT) with the relative abundance of ≥0.01% in most samples and ≥1% in some samples; (3) always rare taxa (ART) with the relative abundance of <0.01% in all samples; (4) conditionally rare taxa (CRT) with the relative abundance of <0.01% in some samples, but whose abundance is never ≥1%; (5) moderate taxa (MT) with the relative abundance between 0.01% and 1% in all samples; and (6) conditionally rare and abundant taxa (CRAT) with the relative abundance ranging from rare (<0.01%) to abundant (≥1%). For the comparative study of abundant taxa (AT) and rare taxa (RT), AT included the always abundant (AAT) and conditionally abundant taxa (CAT), and RT included always rare taxa (ART) and conditionally rare taxa (CAT).

### 2.3. Statistical Analysis

All statistical analyses were mainly calculated in R (http://www.r-project.org/, version 4.1.0 accessed on 25 November 2020). Dominant phyla and alpha- and beta- diversity were assessed employing a Wilcoxon rank sum test. Levin’s niche breadth indices were assessed employing “niche.width” function in spaa package [25,26]. Distance-decay relationships (DDRs) were evaluated as the slopes of linear least-square regression for the relationships between geographic distance or environmental distance versus community dissimilarity (1-the Bray–Curtis distance). Geographical distance between environmental sites was estimated from the sampling coordinates. The separate effects of geographical distance and environmental factors were evaluated employing partial Mantel and Mantel tests [27]. Environmental factors (excluding pH) were log(x + 1)-transformed to enhance normality and decrease nonlinearity. To avoid strong collinearity between the factors, all environmental factors were tested employing “vif.cca” function in vegan package, excluding those factors with variance expansion factor (VIF) greater than 10 [28]. Significant environmental factors were adopted to carry out a distance-based redundancy analysis (db-RDA) employing “capscale” function in vegan package. Variation partitioning analysis (VPA) was employed to estimate the relative impacts of geographic or environmental variables, with the significant geographic or environmental factors as the explanatory variable and Bray–Curtis dissimilarity matrix as the response variable. To assess the assembly processes, we used the normalized stochasticity ratio (NST) assessed in the NST package and nearest taxon index (NTI) assessed in picante package [29]. NST values were estimated based on the Jaccard and Bray–Curtis distances were used to investigate assembly processes, NST is a newly developed indicator with 0.5 as the threshold between more stochastic (>0.5) and more deterministic (<0.5) assembly processes (Ning et al., 2019). Generally, NTI values > +2 and <−2 indicate that assembly was controlled by deterministic and stochastic processes, respectively.

We retained the OTUs detected in at least one fifth of 36 rhizosphere soil samples to improve statistical confidence [20,30]. Spearman rank correlation coefficients between OTUs were estimated in Hmisc package [31]. Spearman correlations (|r| > 0.6) and FDRp < 0.01 were retained in the ecological networks [32]. We performed network visualization using the Gephi software (https://gephi.org/, version 0.9.2 accessed on 25 November 2020, Mathieu Bastian and Sebastien Heymann, Paris, France). The major modules (namely, top four major modules with the greatest mean abundance) in the broomcorn millet bacterial and fungal networks also were selected and analyzed using the Gephi software. We also performed Spearman correlation analysis using the trimmed means of M (TMM) normalized counts per million (CPM) counts of major modules versus environmental variables (soil pH and MAT). Subsequently, we estimated degree, closeness centrality, betweenness centrality, and eigenvector centrality using the igraph package.

## 3. Results

### 3.1. General Distribution of Rare and Abundant Taxa

Based on the lowest number of sequence reads within all samples, the fungal and bacterial sequences were refined to a uniform depth of 43,991 and 28,025 per sample, and they were clustered into 2737 fungal OTUs and 8457 bacterial OTUs based on 97% similarity.

Generally, the abundant taxa (AT) of bacteria and fungi contained 30 and 17 OTUs, which accounted for 0.38% and 0.62% of total OTUs, while 8227 and 2623 OTUs (97.28% and 95.84% of total OTUs) were considered to be rare taxa (RT) of bacteria and fungi, respectively. Furthermore, the moderate taxa (MT) of bacteria and fungi constituted 190 and 2 OTUs, which accounted for 2.25% and 0.07% of total OTUs, respectively. The conditionally rare and abundant taxa (CRAT) included 10 and 95 OTUs, accounting for 0.12% and 3.47% of the total OTUs, respectively (Table 1).

**Figure 1:**
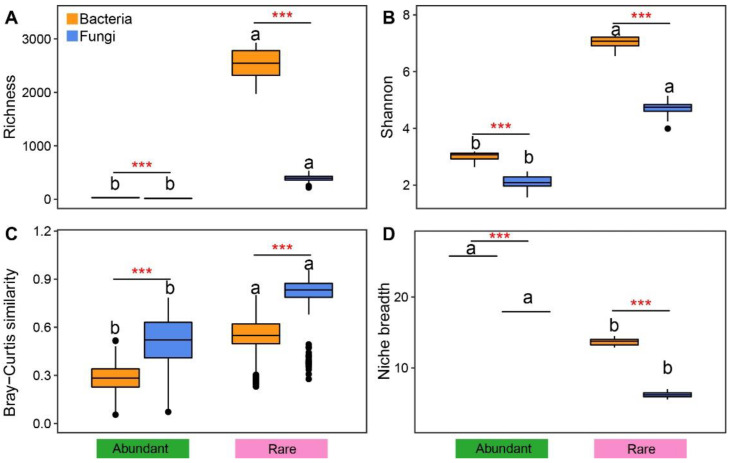
Variations in diversity and niche breadth of abundant and rare rhizosphere bacteria and fungi in broomcorn millet field. (**A**) Richness box plot; (**B**) Shannon box plot; (**C**) Bray–Curtis dissimilarity box plot; (**D**) niche breadth box plot. Different letters above bars and *** indicate significant differences (*p* < 0.05, and <0.001) according to the nonparametric Mann–Whitney U test.

Normally, the Shannon and Richness indicators of rare taxa of rhizosphere fungi and bacteria had considerably greater values than those of abundant ones, and the average values of Shannon and Richness indicators were obviously greater for bacterial rare and abundant than for fungal ones (*p* < 0.001, Figure 1A,B). Similarly, the fungal and bacterial abundant taxa showed smaller beta-diversity than that of rare ones (Figure 1C). The fungal rare and abundant taxa had greater beta diversity than those of the rare ones of bacteria. We also noted that the fungal and bacterial abundant taxa showed higher niche breadth values than those of rare taxa (Figure 1D). There were significant differences in the relative abundances of phylum and family levels between the fungal and bacterial rare and abundant taxa (Figure S2A,B). Actinobacteria, Proteobacteria, and Firmicutes of bacterial abundant taxa, and Mortierellomycota of fungal abundant taxa were considerably greater than those of rare taxa (Figure S2A), and Micrococcaceae, Sphingomonadaceae, and Geodermatophilaceae of bacterial abundant taxa and Nectriaceae, Chaetomiaceae, and Mortierellaceae of fungal abundant taxa also were considerably higher than those of rare taxa (Figure S2B). We also noted that the phyla level classifications of bacteria and fungi are mainly Actinobacteria, Proteobacteria, and Mortierellomycota, and family level classifications of bacteria and fungi are mainly Sphingomonadaceae and Nectriaceae (Table S1).

### 3.2. Patterns and Driving Factors of Beta-Diversity in Abundant and Rare Taxa

**Figure 2:**
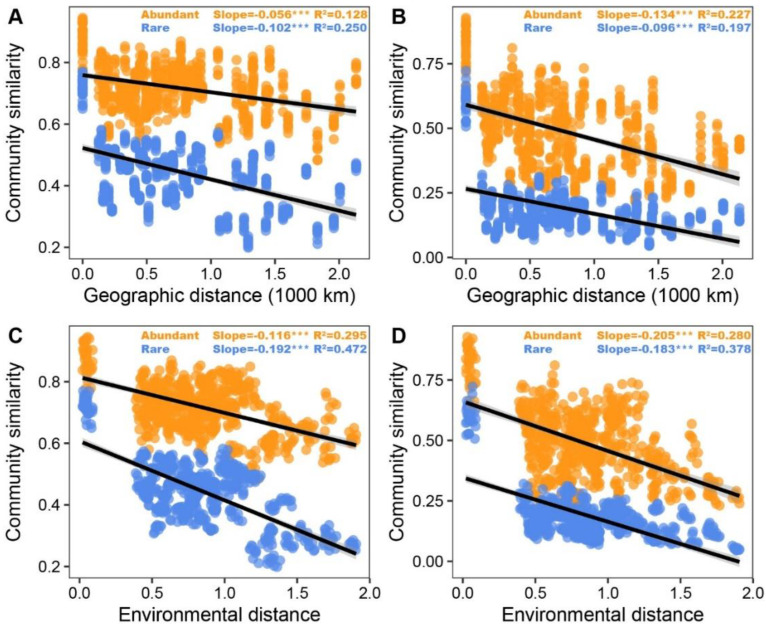
Distance–decay curve of changes in geographic distance (**A**,**B**) and environmental variables (**C**,**D**) versus rhizosphere bacterial (**A**,**C**) and fungal (**B**,**D**) β-diversity of the abundant and rare taxa. ***, *p* < 0.001.

**Figure 3:**
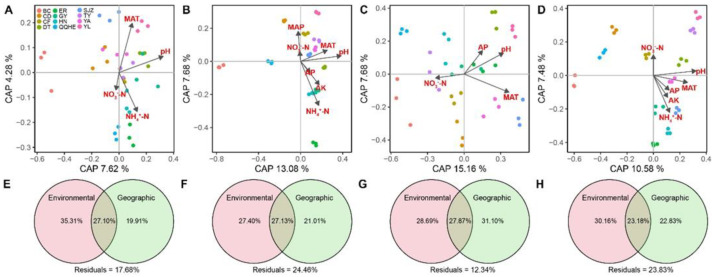
Effects of environmental variables on rhizosphere bacterial and fungal community turnover of the abundant and rare taxa in broomcorn millet fields. Constrained analysis of principal coordinates model showing significant factors that affecting rhizosphere bacterial (**A**,**B**) and fungal (**C**,**D**) community turnover of the abundant and rare taxa. Variation partitioning (% variation explained) of rhizosphere bacterial (**E**,**F**) and fungal (**G**,**H**) community turnover of the abundant and rare taxa in relation to combined environmental and geographic distance.

As revealed by DDRs, the slopes for community similarity versus geographic and environmental distance were steeper for bacterial rare taxa (−0.102 and −0.192, respectively, *p* < 0.001) than for bacterial abundant taxa (−0.056 and −0.116, respectively, *p* < 0.001), while the opposite trend was noted for fungal taxa. Moreover, the slope for community similarity versus environmental distance of fungal and bacterial abundant and rare taxa were steeper than the slope for community similarity versus geographic distance (Figure 2). Mantel test also indicated that the slopes of bacterial rare taxa dissimilarity for both environmental and geographic distance were steeper than those of bacterial abundant taxa dissimilarity, whereas the slopes of fungal abundant taxa for geographic distance were steeper than those of rare taxa dissimilarity; this phenomenon was not found in the slopes of fungal rare taxa dissimilarity for environmental distance (Table S2). Not surprisingly, these results were also confirmed by VPA (Figure 3E–H).

We used a linear regression model according to constrained analysis of principal coordinates (CAP) with forward selection to select the crucial environmental factors driving the fungal and bacterial rare and abundant taxa (Figure 3A–D). Our results indicated that four, seven, four, and six environmental variables were obviously associated with the structure of fungal and bacterial rare and abundant taxa (Figure 3). Among them, MAT and soil pH were the key drivers that jointly regulated the variations in the fungal and bacterial abundant and rare taxa. A similar phenomenon was confirmed by Mantel tests (Table 2).

### 3.3. Assembly Processes in Abundant and Rare Taxa

**Figure 4:**
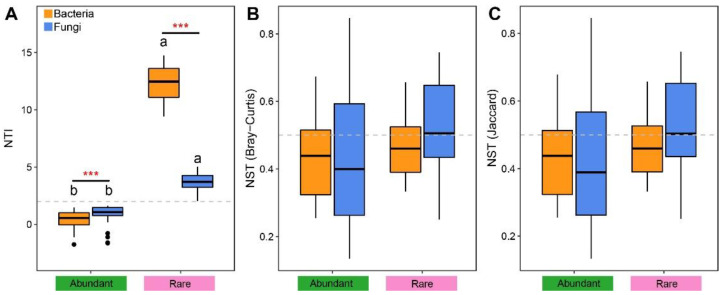
The nearest taxon index (NTI) (**A**) and normalized stochasticity ratio (NST) based on Bray–Curtis distance (**B**) and Jaccard distance (**C**) of abundant and rare rhizosphere bacterial and fungal subcommunity. Different letters above bars and *** indicate a significant difference at the *p* < 0.05 and 0.001 level according to nonparametric Mann–Whitney U test.

The relative impacts of deterministic and stochastic processes to governing the taxa in both fungal and bacterial community composition was estimated using the normalized stochasticity ratio (NST) and nearest taxon index (NTI) (Figure 4). We noted that the NTI mean values of abundant and rare taxa of fungi and bacteria were greater than zero. In addition, all fungal and bacterial rare taxa were phylogenetically clustered together (NTI > 2) (Figure 4A). These results were confirmed by NST-based community assembly analysis (Figure 4B,C), and the abundant and rare fungi and abundant bacteria were shown to be governed by deterministic processes.

### 3.4. Co-Occurrence Patterns of Abundant and Rare Taxa

**Figure 5:**
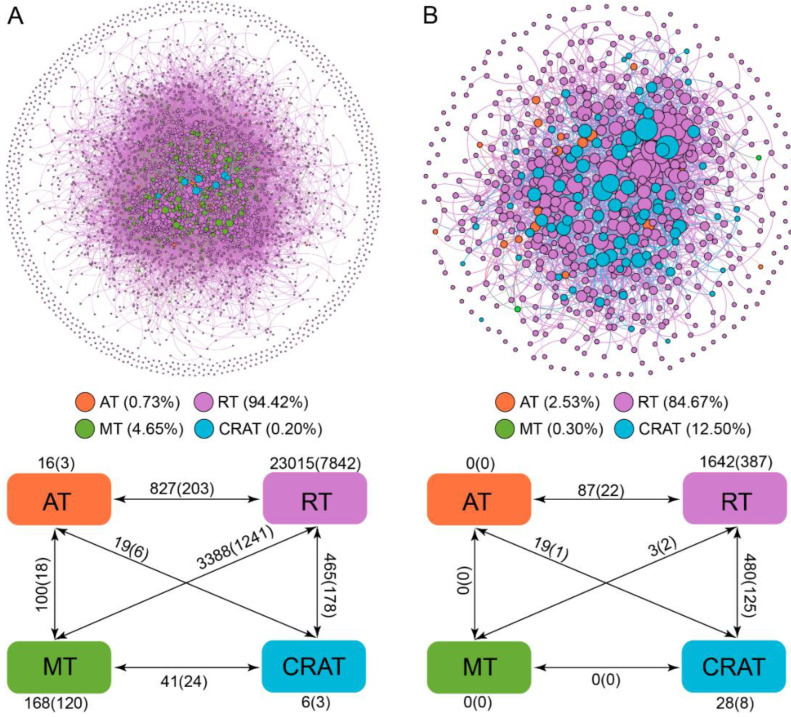
Co-occurring network of rhizosphere bacterial (**A**) and fungal (**B**) community within each subcommunities based on correlation analysis. The nodes in network are colored by the category of subcommunities. The size of each node in proportion to the number of connections (i.e., degree). The connections in the network indicate strong (Spearman’s r > 0.6 or <−0.6) and significant (*p* < 0.01) correlations. Numbers outside and inside parentheses represent the numbers of positive and negative edges, respectively. AT, abundant taxa (orange); RT, rare taxa (purple); MT, moderate taxa (green); CRAT, conditionally rare and abundant taxa (sky blue).

The fungal and bacterial networks contained 672 and 4088 nodes linked by 2804 and 37,683 edges, respectively. Among the nodes, the fungal and bacterial abundant taxa accounted for a small proportion of total number of OTUs (2.53% and 0.73%, respectively), whereas rare taxa accounted for the most of total number of OTUs (84.67% and 94.42%) (Figure 5). The fungal and bacterial networks exhibited scale-free characteristics (Figure S3), indicating that it is nonrandom. The observed ACC, APL, and modularity of fungal and bacterial networks were higher than those of respective random networks, indicating that the networks had “small-world” characters and modular structure (Table S3). Not surprisingly, the non-rare taxa (MT, AT, and CRAT) had a stronger interaction with RT than with themselves (Figure 5).

Generally, the node-level topological characteristics of fungal and bacterial rare taxa were similar to those of abundant taxa (Figure S4A–D). Both the values of degree and betweenness centrality of conditionally rare and abundant taxa of fungi and bacteria were considerably greater than those of rare taxa (*p* < 0.05, Figure S4A,B). Furthermore, considerably lower closeness centrality values were observed for rare taxa of fungi than for conditionally rare and abundant taxa, but similar phenomena were not found in bacteria (Figure S4D). However, eigenvector centrality values between the four taxa were not significantly different (Figure S4C). The values of degree and betweenness centrality of bacterial rare taxa were considerably greater than those of fungal rare taxa (Figure S4A,B).

**Figure 6:**
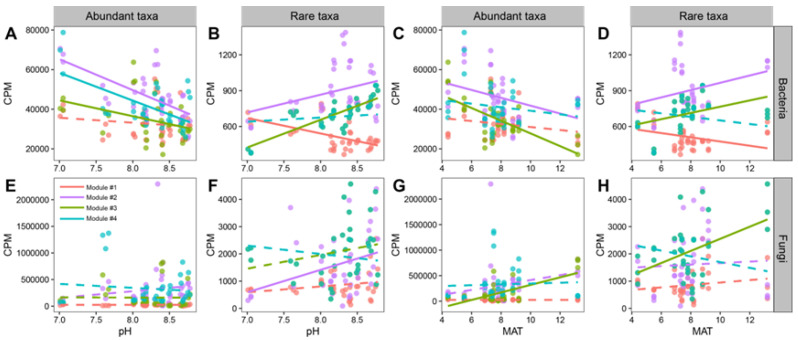
Regression analysis between selected environmental factors (pH and MAT) and accumulated relative abundance of rare and abundant bacterial (**A**–**D**) and fungal (**E**–**H**) modules in the co-occurrence network. Solid lines indicated significant relationships of *p* < 0.05; dashed for *p* > 0.05.

The fungal and bacterial networks were divided into four major modules, which accounted for 53.56% and 68.86% of the entire network, respectively. Across the four fungal and bacterial modules, rare taxa accounted for a great proportion, whereas abundant taxa accounted for only a small proportion (Figure S5). In Modules #1, #2, #3, and #4, the family level compositions of rare bacteria are richer than that of abundant bacteria. In Module #1, #2, #3, and #4, the relative abundance of Nectriaceae in abundant taxa is higher than that of rare taxa (Table S4). Abundant and rare taxa of bacteria were significantly associated with pH and MAT, especially in Modules #2 and #3. The abundant taxa of Modules #2 and #3 were negatively associated with pH and MAT, while the opposite trends were found for the rare taxa (Figure 6A–D). Meanwhile, the rare taxa of Module #1 were negatively correlated with pH and MAT (Figure 6B,D). The rare fungal taxa of Module #2 were positively correlated with pH (Figure 6F). Rare and abundant taxa of Module #3 were positively related to MAT (Figure 6G,H).

## 4. Discussion

### 4.1. Distribution Patterns of Abundant and Rare Microbes

In the present study, rare taxa of fungi and bacteria exhibited greater alpha diversity than that of abundant taxa (Figure 1), indicating rare taxa are the essential contributors to the diversities of the entire microbial community [33,34]. This was consistent with the results of previous similar reports in various ecosystems, including marshes, oceans, contaminated soils, and terrestrial farmland [16,17,35,36]. The subjects of these studies had great environmental heterogeneity. Therefore, it could be concluded that rare taxa are more fragile than abundant taxa. This phenomenon can be explained by the following notions: (i) abundant taxa have broader niches than rare ones (Figure 1D), and they thereby competitively utilize resources to maintain their persistence; (ii) rare taxa, which have many metabolically diverse organisms, can respond quickly to environmental variations [37]; and (iii) rare taxa are more likely to be extinct, and sequencing methods might only detect a few rare taxa, which limits their distribution [17]. Furthermore, we found that fungal and bacterial rare taxa also exhibited greater beta-diversity than that of abundant taxa (Figure 1C), which might indicate that external microorganisms are imported through diffusion [6]. Microbial communities are the major contributors to ecosystem functions [38]. We found that taxonomic distribution of fungal and bacterial abundant and rare taxa was substantially distinct. Furthermore, we observed that the relative abundances of Actinobacteria, Proteobacteria, Acidobacteriota, Chloroflexi, and Mortierellomycota exhibited obvious differences between rare and abundant taxa, which was in line with the findings of studies on both terrestrial and aquatic ecosystems [35,39,40,41]. These results collectively confirmed that rare and abundant taxa in various ecosystems have similar species classifications [16].

### 4.2. Biogeographic Patterns of Abundant and Rare Microbes

The biogeographic microbial pattern might be an essential index for assessing the environmental health of complex ecosystems; thus, disentangling the mechanisms understanding this pattern is a vital subject in microbial ecology [42,43]. We noted that fungal and bacterial abundant and rare taxa exhibited robust DDRs across spatial and environmental scales in the investigated proso millet fields. However, bacterial rare taxa had a greater phylogenetic turnover rate (steeper slope of DDRs) than bacterial abundant ones did, revealing that rare taxa of bacteria were assembled non-randomly, and their members were exhibited in a narrow geographic range (Figure 2D). The opposite trend was observed in fungal taxa. This was in line with previous findings on bacterial communities in forest soil [44] and fungal communities in wetland soils [45]. Moreover, the slopes of fungal and bacterial rare and abundant taxa for community similarity versus environmental distance were steeper than those for community similarity versus spatial distance (Figure 2). The VPAs and Mantel test together confirmed that, compared to spatial distance, environmental factors better explained the variation in fungal and bacterial beta-diversity (excluding abundant fungi) (Figure 3 and Table S2). These results revealed that fungal and bacterial rare and abundant taxa showed distinct biogeographic distribution patterns in the proso millet fields. Similar results have also been reported in previous literature revealing that environmental factors (such as soil pH, water, and nutrient availability) are essential variables affecting the distribution patterns of soil microbial communities in various ecosystems [46,47,48,49]. We speculated that environmental selection may be more stringent and become even more pronounced in governing the biogeographic patterns of microbial communities [50]. Additionally, the great differences in soil properties among sampling sites offered a wider range of environmental gradients, which was conducive to the environmental filtration of microbial communities [51].

In our study, we also highlighted the primary impacts of environmental variables on the community structures of bacteria and fungi across northern China. Of all the environmental variables measured, soil pH and MAT had the highest effect on the structure of fungal and bacterial rare and abundant taxa (Figure 3A and Table 2). Our findings were similar to those of previous reports, and they offered solid evidence that these drivers have an obvious influence on abundant and rare taxa [35,44]. We suggested that these environmental drivers should exert strong selection pressures on the microbial taxa in proso millet fields.

### 4.3. Assembly Processes of Abundant and Rare Microbes

A core debate in biogeography is how to balance the relative impacts of deterministic and stochastic processes on the community assembly [52]. In our study, the mean NTIs of fungal and bacterial abundant and rare taxa were obviously greater than zero (Figure 4A), indicating that these taxa were phylogenetically clustered more closely than expected, and confirming that deterministic processes are crucial in dominating the fungal and bacterial community assembly at a regional scale [53], which was corroborated by NST analysis (Figure 4B,C). This is agreed with the results of several literature exhibiting the assembly of bacterial rare and abundant being affected by variable selection [45,54], while homogeneous selection shaped the community assembly of rare fungi [35]; on the other hand, our findings differed from previous observations indicating that dispersal limitation governed bacterial abundant taxa as well as fungal abundant and rare taxa [45]. These differences might be related to variability in habitat properties and geographical conditions [55]. We also confirmed that the NTI values of bacterial rare taxa were obviously greater than those of fungal rare taxa, while the opposite trend was noted for abundant taxa (Figure 4A). On the one hand, difference in the assembly process of fungal and bacterial rare and abundant taxa may be a consequence of environmental heterogeneity and the ability of different taxa to adapt to environmental variations [56,57]. On the other hand, it might also be related to discrepancies in the life cycles and cell sizes of bacteria and fungi, which are generally considered to be important factors impacting the dispersal potential of organisms [58,59]. Therefore, metabolic activities and dispersal abilities may influence the role of determinism or stochasticity in shaping community composition [45,60,61]. Based on the above results, we concluded that fungal and bacterial rare and abundant taxa occupied distinct ecological niches and played major roles in the investigated proso millet field.

### 4.4. Co-Occurrence Patterns of Abundant and Rare Microbes

Identifying the ecologic patterns of rare and abundant taxa could enhance our cognition of microbial interactions [62]. The results showed that the investigated communities had non-randomly connected characteristics, a power-law distribution. Generally, active interactions in a network can indicate cooperation [13]. Most positive interactions were found among rare taxa and between rare taxa and other taxa, which might contribute to the network species separation, modularization, and ecosystem function stability [63,64,65]. One study has confirmed that less abundant taxa can serve as essential key taxa in the networks [62], and that the removal of these key species could lead to disruption of microbial networks, indicating that rare taxa might be just as crucial as abundant taxa in keeping ecosystem functions [62]. We also found that, compared to abundant taxa, rare taxa had greater degree values but smaller betweenness centrality values, although there were no significant differences; this was especially true for fungi. Overall, rare taxa played an essential role in keeping ecosystem functions.

In network analysis, dividing the network into modules offers insight into distinct clusters of nodes that carry out distinct ecosystem functions [66]. We divided the four major modules, and they were mainly composed of rare taxa, revealing their important roles in community assembly [62]. Accordingly, changes in rare taxa might cause the sharp variations in community modules and networks [67]. Moreover, we investigated the relationship of the main fungal and bacterial modules with environmental variables (such as MAT and soil pH), and we noted that MAT and soil pH were considerably related to the modules of fungal and bacterial rare and abundant taxa (Figure 6), which was in accordance with the results found in previous literature [68,69].

## 5. Conclusions

Through large-scale sampling of proso millet farmland soils in northern China, we investigated the ecology of fungal and bacterial community distribution by explored the rare and abundant taxa. In addition to having higher diversity, the rare taxa of fungi and bacteria also showed obviously stronger phylogenetic clustering than abundant taxa. Furthermore, bacterial rare taxa exhibited a greater phylogenetic turnover rate than abundant taxa, whereas the opposite trend was noted in fungal taxa. Environmental variables, especially MAT and soil pH, were the essential variables affecting the structure of fungal and bacterial rare and abundant taxa. The assembly of rare and abundant taxa was mainly governed by deterministic processes. The network analysis suggested that fungal and bacterial rare taxa play a major role in keeping ecosystem functions. Meanwhile, pH and MAT were also important factors governing the major modules of abundant and rare taxa. Altogether, this study offers a new perspective on the ecological significance and biogeographic distribution of rare and abundant taxa in proso millet fields, reinforcing recent findings on fungal and bacterial microbial interactions.

## Figures and Tables

**Table 1:** Identification and information of abundant and rare bacteria and fungi.

		Abundant Taxa (AT)	Rare Taxa (RT)	Moderate Taxa (MT)	Conditionally Rare and Abundant Taxa (CRAT)
OTU number (%)	Bacteria	30 (0.38%)	8227 (97.28%)	190 (2.25%)	10 (0.12%)
	Fungi	17 (0.62%)	2623 (95.84%)	2 (0.07%)	95 (3.47%)

**Table 2:** Mantel tests based on Spearman’s rank correlations show the relationships between the bacterial and fungal community structures and environmental variables.

	Bacteria		Fungi	
	Abundant	Rare	Abundant	Rare
pH	0.432 ***	0.525 ***	0.392 ***	0.431 ***
OM	0.267 **	0.257 **	0.331 ***	0.232 **
TN	0.278 ***	0.258 **	0.203 **	0.234 ***
AP	0.189 *	0.125	0.105	0.177 *
AK	−0.064	−0.050	0.060	0.013
NH_4_^+^-N	0.065	0.252 ***	−0.016	0.275 ***
NO_3_^−^-N	0.026	0.048	0.229 **	0.158 *
MAT	0.478 ***	0.449 ***	0.592 ***	0.541 ***
MAP	0.032	0.074	0.087	0.132 *

Note: *, *p* < 0.05; **, *p* < 0.01; ***, *p* < 0.001.

## Data Availability

The fungal and bacterial raw sequence data can be gained in the NCBI Sequence Read Archive under PRJNA772160 and PRJNA772155, respectively.

## Supplementary Materials

The following are available online at https://www.mdpi.com/article/10.3390/microorganisms11010134/s1. Table S1 Taxonomic identification of abundant bacterial and fungal communities; Table S2 The relationship between community dissimilarity and the environmental distance or geographic distance using Mantel and partial Mantel tests; Table S3 Key topological features of the microbial networks among three minor grain crop rhizosphere soils; Table S4 Class level composition of abundant and rare taxa of bacteria and fungi in the four main modules; Figure S1 Location of the twelve sampling sites of broomcorn millet crops in agricultural fields; Figure S2 Taxonomic distribution of the rare and abundant bacterial and fungal taxa at the phylum (A) and family (B) level in broomcorn millet soils. ns—not significant, * *p* < 0.05, *** *p* < 0.001 (Wilcoxon rank sum tests); Figure S3 Relative influences of assembly processes governing the abundant and rare taxa of rhizosphere bacteria (A) and fungi (B). The solid black line represents the 95% confidence interval above and below the prediction; Figure S4 Degree distribution for the bacterial (A) and fungal (B) co-occurrence networks in the broomcorn millet rhizosphere; Figure S5 Bar charts showing the richness proportion of different subcommunities in each major module of the co-occurrence network. Abundant: abundant bacterial and fungal subcommunity. Rare: rare bacterial and fungal subcommunity. Other, other bacterial and fungal subcommunity [20,21,22,70].
