# Supplementary material for: Biogeographic Pattern and Network of Rhizosphere Fungal and Bacterial Communities in Panicum miliaceum Fields: Roles of Abundant and Rare Taxa
Source: Microorganisms. 2023 Jan 4;11(1):134. doi: 10.3390/microorganisms11010134 (PMC9863577; doi:10.3390/microorganisms11010134)
Supplement: Supplementary file 1 [file microorganisms-11-00134-s001.zip › microorganisms-2039474-supplementary.pdf]

Research paper

Biogeographic pattern and network of rhizosphere fungal and bacterial communities in *Panicum miliaceum* fields: roles of abundant and rare taxa

Lixin Tian[1], Feifei Zhang[1], Liyuan Zhang[2], Lei Zhang[3], Xiaoli Gao[1*], Baili Feng[1*]

[1]*College of Agronomy/State Key Laboratory of Crop Stress Biology in Arid Areas, Ministry of Agriculture, Northwest A & F University, Yangling, Shaanxi 712100*

[2]*Chifeng Academy of Agricultural and Animal Husbandry Sciences, Chifeng, Inner Mongolia 024031*

[3]*Crop Research Institute, Gansu Academy of Agricultural Sciences, Lanzhou, Gansu 730070*

* Corresponding author: Prof. Baili Feng and Prof. Xiaoli Gao. State Key Laboratory of Crop Stress Biology in Arid Areas, Ministry of Agriculture, College of Agronomy, Northwest A & F University, Yangling, Shaanxi 712100, PR China

E–mail: fengbaili@nwafu.edu.cn (BL Feng) and gao2123@nwsuaf.edu.cn (XL Gao)

Phone: +86–29–87082889

Fax: +86–29–87082889

2. Materials and Methods

2.1 Soil sample analysis

All samples were sieved through a < 2 mm sieve. A subset of soil samples was air-dried and analysed for edaphic properties, the analysed parameters included pH, organic matter (OM), total nitrogen (TN), available potassium (AK), available phosphorus (AP), nitrate (NO_3^--N), and ammonium (NH_4^+-N).

Briefly, soil pH was measured in a soil-water slurry (1:2.5, soil/water ratio) using a PHS-3C pH meter (Shanghai INESA Scientific Instrument Co., Ltd, Shanghai, China). Soil organic matter (OM) was measured using the potassium dichromate ($K_2Cr_2O_7$) oxidation method. Soil total nitrogen (TN) was determined by the Kjeldahl digestion method. Soil available potassium (AK) was determined using neutral ammonium acetate extraction and analysis by flame photometry. Soil available phosphorus (AP) was determined using $NaHCO_3$ solution extraction and analysis by Mo-Sb colorimetric method. Soil samples were shaken for 1 h in 2 M KCl (5 g soil in a 50-ml KCl solution) and then filtered to analyze the concentrations of soil nitrate (NO_3^--N), ammonium (NH_4^+-N) with an auto analyzer (SEAL-AA3, Germany).

2.2 DNA extraction and sequence analysis

According to the statements of Tian et al. (2022), the DNeasy 96 PowerSoil Pro QIAcube HT kit (QIAGEN, Hilden, Germany) was used to extract total DNA from 500 mg soil in each sample. The total DNA was submitted to Majorbio Bio-pharm Technology Co., Ltd (Shanghai, China) for high-throughput sequencing on the MiSeq PE300 platform (Illumina, San Diego, CA, USA). The V3–V4 regions of bacterial 16S rRNA genes in the

rhizosphere samples were amplified using 338F (5'-ACTCCTACGGGAGGCAGCA-3') and 806R (5'-GGACTACHVGGGTWTCTAAT-3') primer sets with unique barcodes. The fungal ITS1 region in the rhizosphere samples was amplified using ITS1F (5'-CTTGGTCATTTAGAGGAAGTAA-3') and ITS2R (5'-GCTGCGTTCTTCATCGATGC-3') primer sets with unique barcodes. The obtained raw sequences were filtered for quality control, and chimeric sequences were removed using USEARCH version 7.0 (http://www.drive5.com/usearch) (Edgar et al. 2011). All sequences were merged and assigned to operational taxonomic units (OTUs) at a 97% similarity criterion using the UPARSE algorithm (https://drive5.com/usearch/manual/pipe_otus.html) (Edgar et al. 2011). The bacterial and fungal OTU sequences were aligned against the Silva database (release 138 https://www.arb-silva.de/) (Quast et al. 2013), and the Unite database (Release 8.0 http://unite.ut.ee/index.php) (Nilsson et al. 2019), respectively.

References

Edgar, R.C., Haas, B.J., Clemente, J.C., et al., 2011. Uchime improves sensitivity and speed of chimera detection. Bioinformatics 27, 2194-2200.

Nilsson, R.H., Larsson, K., Taylor, A., et al., 2019. The unite database for molecular identification of fungi: handling dark taxa and parallel taxonomic classifications. Nuclc Acids Research 47, D259-D264.

Quast, C., Pruesse, E., Yilmaz, P., et al., 2013. The silva ribosomal rna gene database project: improved data processing and web-based tools. Nucleic Acids Research 41, D590-D596.

Tian, L.X., Chen, P.L., Gao, Z.J., et al., 2022. Deciphering the distinct mechanisms shaping the broomcorn millet rhizosphere bacterial and fungal communities in a typical agricultural ecosystem of northern china. Plant and Soil.

Table S2 The relationship between community dissimilarity and the environmental distance or geographic distance using Mantel and partial Mantel tests.

Effect of	Controlling for	Bacteria		Fungi	
		Abundant	Rare	Abundant	Rare
Environmental distance		0.416***	0.488***	0.404***	0.472***
Geographic distance		0.295***	0.435***	0.477***	0.408***
Environmental distance	Geographic distance	0.358***	0.413***	0.308***	0.398***
Geographic distance	Environmental distance	0.192**	0.343***	0.406***	0.313***

Table S3 Key topological features of the microbial networks among three minor grain crop rhizosphere soils.

	Empirical networks									Random networks		
	Nodes	Edges		APL	ACC	Diameter	Density	Modularity	Degree	APL	ACC	Modularity
		Positive	Negative									
Bacteria	4088	28045	9638	4.49	0.31	13	0.003	0.6	13.72	3.136±0.0002	0.005±0.0001	0.188±0.0027
Fungi	672	2259	545	4.57	0.35	11	0.01	0.6	6.72	3.310±0.0011	0.012±0.0014	0.307±0.0027

APL: average path length; ACC: average clustering coefficient

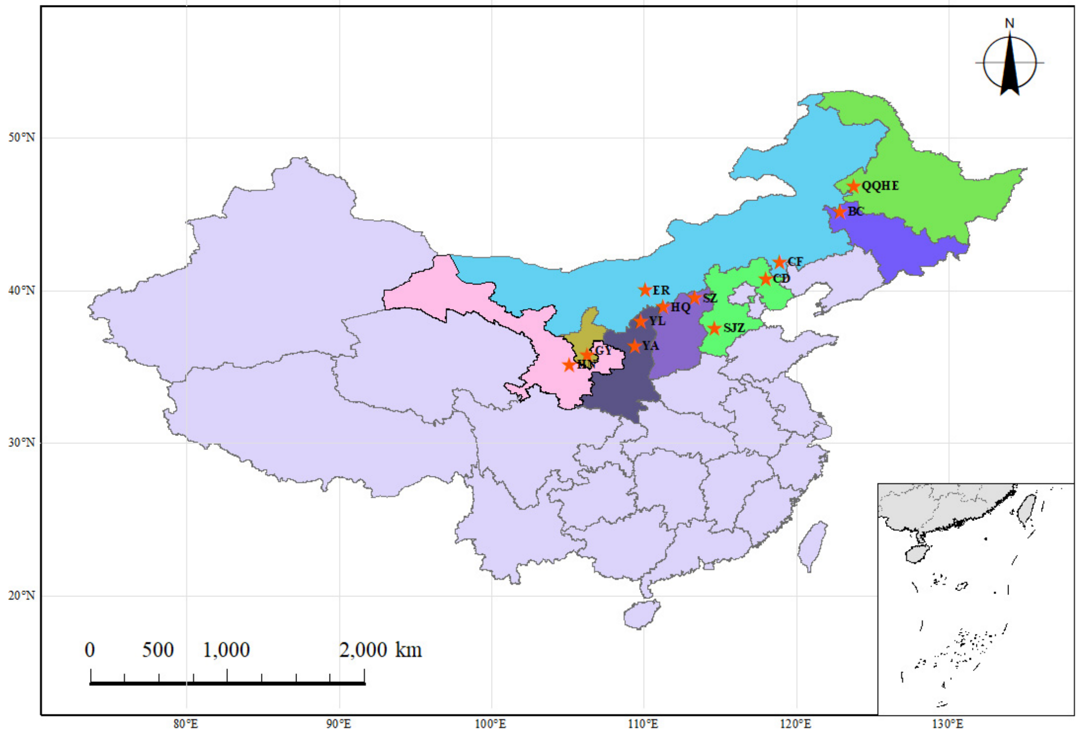

Figure S1 Location of the twelve sampling sites of broomcorn millet crops in agricultural fields

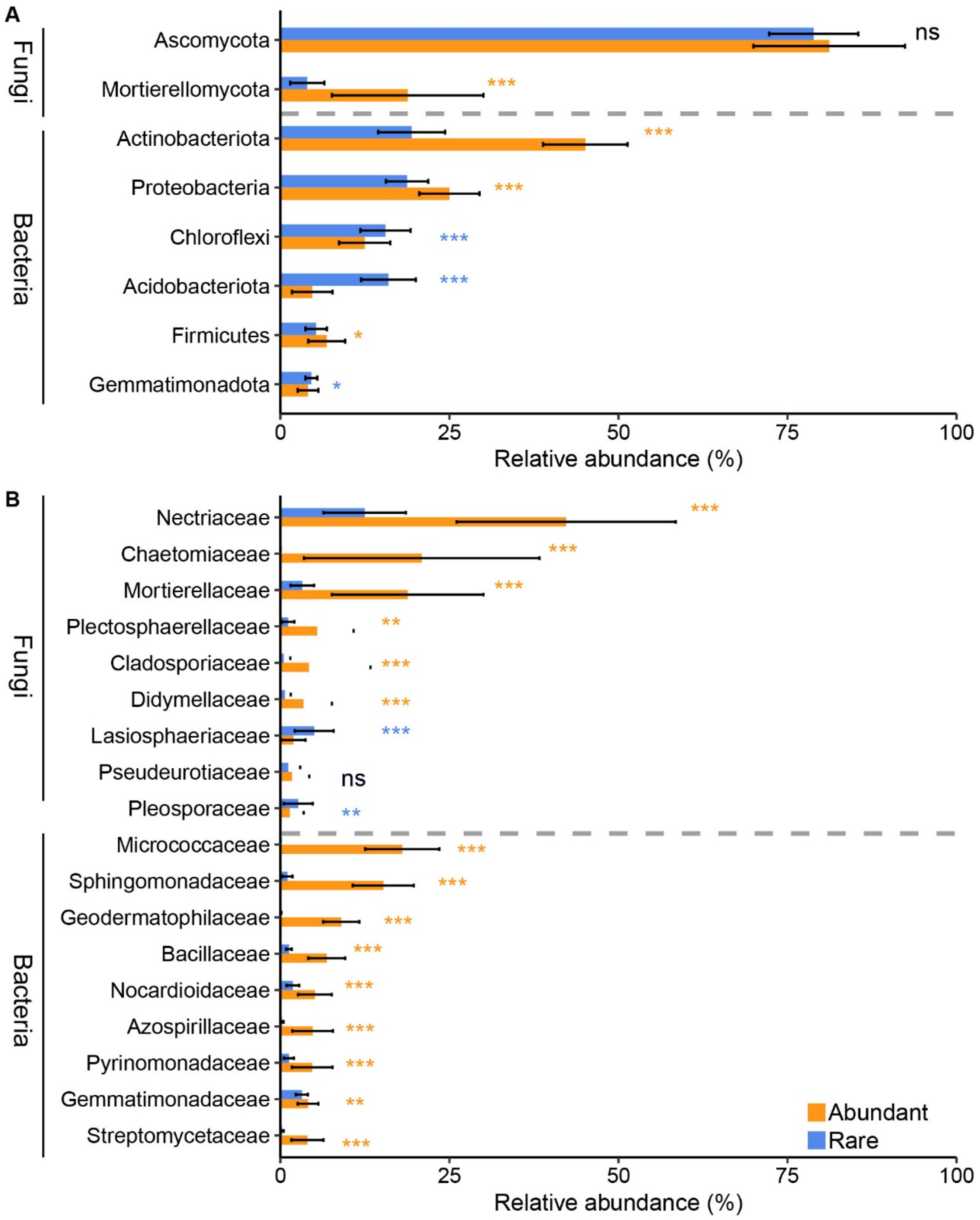

Figure S2 Taxonomic distribution of the rare and abundant bacterial and fungal taxa at the phylum (A) and family level (B) in broomcorn millet soils. ns—not significant, *p < .05, **p < .01, ***p < .001 (Wilcoxon rank sum tests)

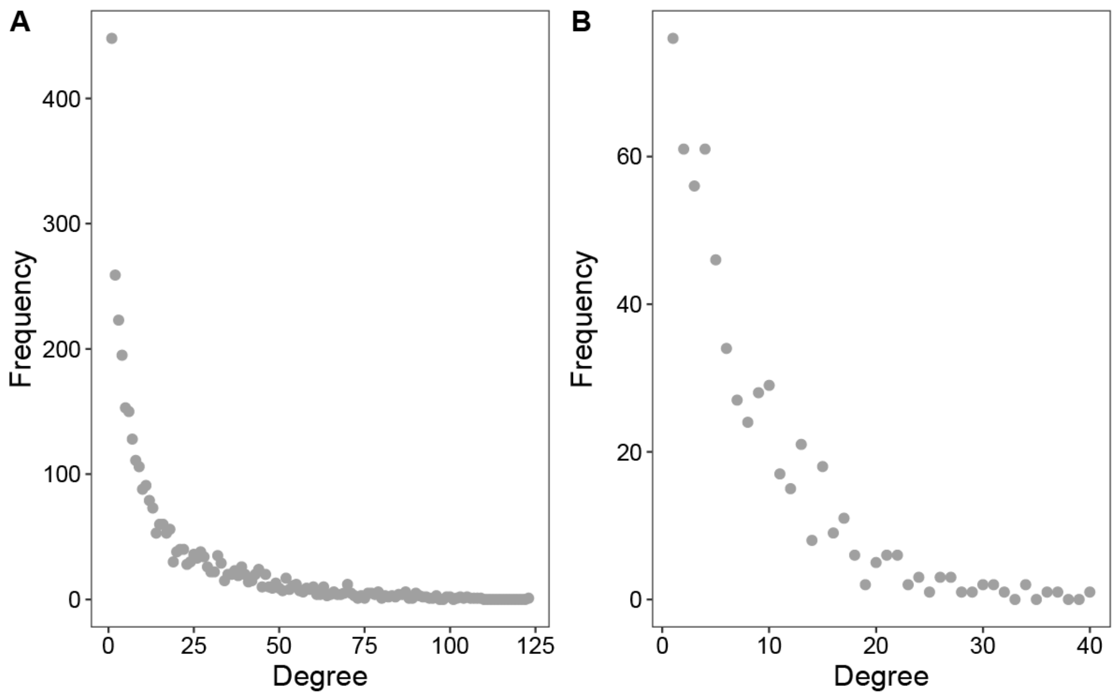

Figure S3 Degree distribution for the bacterial (A) and fungal (B) co-occurrence networks in the broomcorn millet rhizosphere.

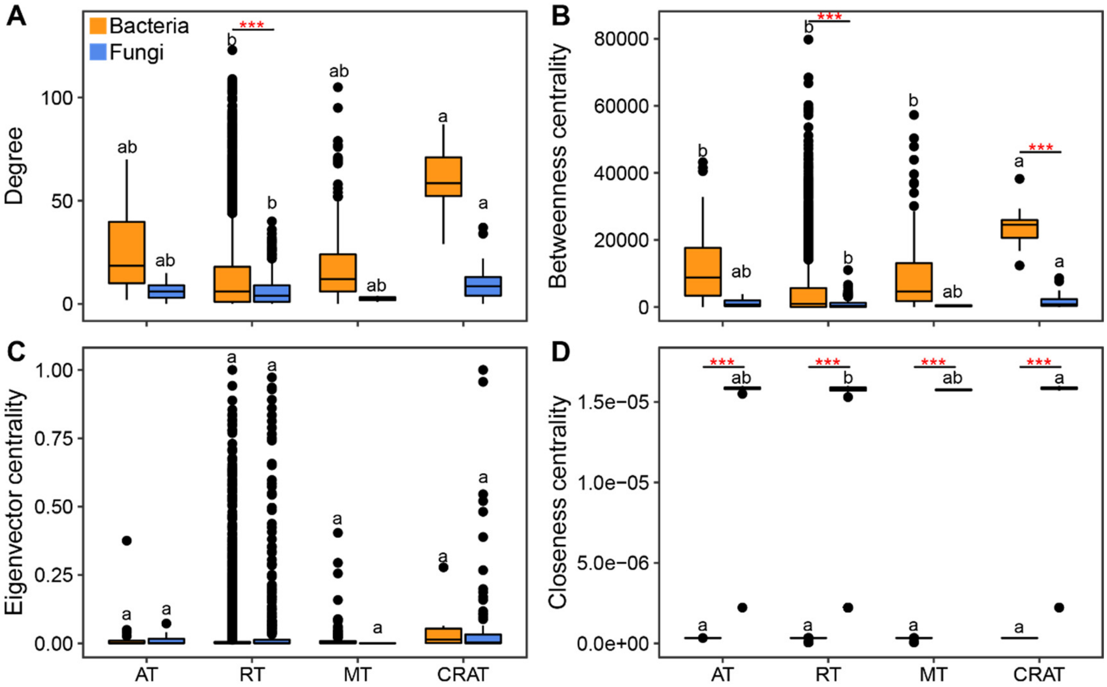

Figure S4. Topological properties of the four subcommunities. *** indicate the significant differences at P < 0.001 based on the Mann-Whitney U test between two groups, respectively. AT, abundant taxa; RT, rare taxa; MT, moderate taxa; CRAT, conditionally rare and abundant taxa. Different letters above bars indicate significant differences (P<0.05) according to the nonparametric Mann-Whitney U test.

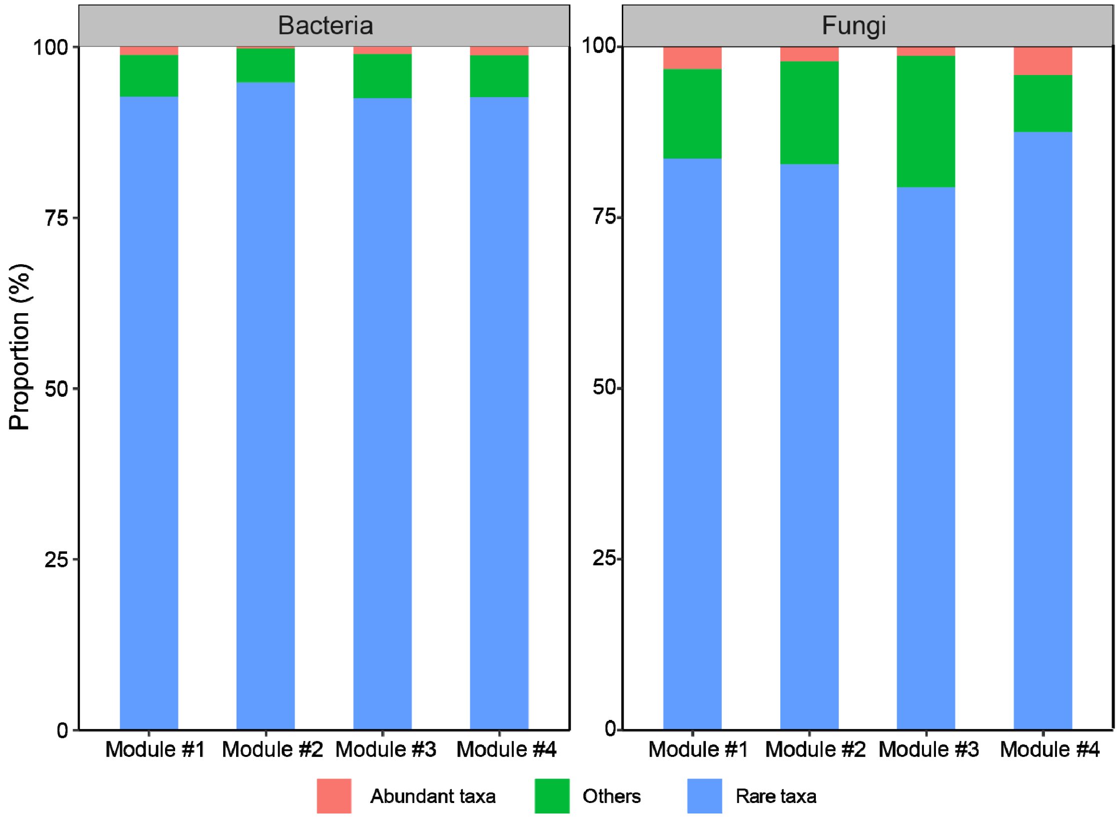

Figure S5 Bar charts showing the richness proportion of different subcommunities in each major module of the co-occurrence network. Abundant: abundant bacterial and fungal subcommunity. Rare: rare bacterial and fungal subcommunity. Other, other bacterial and fungal subcommunity.
